# Flow signatures and catchment’s attributes for HCA clustering in a hydrologic similarity assessment (Tunisian case)

**DOI:** 10.1038/s41598-023-38608-6

**Published:** 2023-07-26

**Authors:** Rim Chérif, Emna Gargouri-Ellouze

**Affiliations:** 1 LR99ES19 Laboratory of Modelling in Hydraulics and Environment (LMHE), National Engineering School of TUNIS (ENIT), University of Tunis El Manar, BP 37, 1002 Tunis, Tunisia; 2 High Institute of Environmental Sciences and Technology (HIEST), Borj Cedria. Carthage University, Tunis, Tunisia

**Keywords:** Environmental sciences, Hydrology

## Abstract

Partitioning methods such as cluster analysis are advantageous in pooling catchments into hydrometric similar regions. They help overcome data shortage in ungauged catchments, which is a common problem in Sud Mediterranean zones. Without accurate forecasts, it is difficult to assess and manage water resources efficiently this situation won't be of any assistance to hydrology decision-makers. This paper illustrates a Tunisian application case, that aims to pool catchments with a hierarchical clustering algorithm (HCA) based on distances calculated in multidimensional physiographical and hydrometric space. The homogeneity of generated clusters is checked by the silhouette index. Then the distances efficiencies are compared. Nineteen semi-arid Tunisian catchments monitored since 1992 are studied. Twelve physiographical attributes, nine rainfall and streamflow signatures are considered in the HCA with two clusters. Correlation distance provides the most homogeneous clusters. Statistically the: percentage of area affected by anti-erosive practices, percentage of forest cover and catchment area are the most discriminating attributes. However, hydrometrical signatures appear to be irrelevant. These partitions highlight two different hydrological behaviors that must support forecasting. Results are promising in the Sud-Mediterranean case, where the shortage of hydrometrical data is an ongoing problem. They have the advantage of enabling hydrologic forecasting without requiring heavy information.

## Introduction

Water resources management (ex: land use planning, irrigation, hydraulic structure design, flood forecasting) requires knowledge of water quantity at a target site or catchment. Nevertheless, several catchments in many parts of the world are ungauged or poorly gauged, this lack of data often increases with decreasing catchment sizes that leads to great difficulties in their management^1,2^. Therefore, runoff prediction at an ungauged river or catchment is carried out through some kind of extrapolation from a gauged site to an ungauged site, and this is not straightforward. This is the whole raison d’être of the Prediction of Ungauged Basin (PUB) initiative^2^. PUB was designed to develop a better scientific basis for hydrology with greater consistency, increasing the prospects for scientific breakthroughs and reducing uncertainties^3^.

Regionalisation techniques are PUB tools that are necessary for transferring information. They belong to two categories; statistical or process based. The transfer of information from one or several gauged catchments (donors) to another ungauged catchment (receiver)^4^ requires the identification of similar gauged catchments, which can be selected through:

- Geographical or spatial proximity.
- Similarities in their hydrologic and/or physiographical and climatic attributes applied with clustering approaches. Thus, Metric distances are commonly identified between catchments in multidimensional attribute space to assess their proximity^5,6^.

In practice, hydrologists explored a large range of approaches for regionalisation over time, as there are no established criteria by which the superiority of any approach can be clearly brought out^7,8^.

Burn and Goel^9^ adopted clustering as a starting point for catchment partitions based on physiographical catchment characteristics with weighted Euclidian distance. Then a regional revision heuristic process was proposed to increase the region’s homogeneity^10^. Lately, Jared et al.^11^ studied, in a classification framework, small Canadian catchments within the Prairie based on climatic and biophysical attributes. They identified similar regions with the agglomerative hierarchical clustering of principal components (HCPC) method. Thus, it can be underlined that regionalisation studies frequently require catchment classification, on which their accuracy closely depends.

Despite the large efforts in PUB, there is still a long way to go in terms of achieving robust and reliable predictions. Ungauged basins have seen less success thus far than gauged basins, which is detrimental to developing countries where the management of sustainable water resources and the development of effective flood and drought mitigation strategies will continue to be hampered by our inability to accurately predict the future^3^.

Unsupervised classification is a data mining technique that is undoubtedly a challenging research area. It could be defined as the organization of a collection of patterns into groups based on similarity analysis^12,13^. Many hydrologic scholars applied this class of clustering algorithms for the purpose of analyzing catchments similarity based on their physiographical, climatic, stream-flow signatures, etc.^14^. Goval and Gupta^15^ divide clustering methods into hierarchical (agglomerative and divisive) and partitional (hard clustering [ex: k-mean] and soft clustering [ex: fuzzy C-mean]).

Partitional clustering methods divide a data set of objects based on their similarity. For K-Means clustering^16^, the number of clusters (K) is defined previously; the initial clusters are first randomly selected, then modified to generate new clusters that minimize the variance within each cluster. Each object can belong to several clusters in the case of soft classification.

Hierarchical cluster analysis (HCA) algorithms pool similar objects into a hierarchy of clusters. They offer a series of interlocked partitions in the form of trees called dendrograms. The main advantage of HCA compared with partitional clustering methods lies in the dendrogram representation, which highlights additional information, such as the increase in dispersion in a cluster generated by an aggregation. It also does not require determining the number of clusters in advance. Indeed, by observing the dendrogram and playing with the depth of the tree, we can explore different possibilities and choose the number of clusters that suit our application case best. Thus, it is conceptually simple, good for small data sets, and less sensitive to noise in the data set^17^.

In our case, HCA is better suited to identify catchment clusters with similar hydrologic behaviors. Metrics (or distances) are used to measure this similarity^9^. Hence, distances evaluate the proximity, or relevance, of each gauged catchment to the target location and identify the most hydrologically similar one^18^. Since it aims to reduce the variance between entities within a cluster, we use it in conjunction with Ward's linkage method^19^.

Many useful distances, such as Euclidean, squared Euclidean, Manhattan, Chebyshev, cosine, Canberra, Minkowski, and Mahalanobis, were cited in the literature. Nathan and Mc Mahon^20^ compared combinations of similarity measures (Euclidean, squared Euclidean, Manhattan, Chebyshev, and Cosine) and linkage methods (simple, complete, average, and Ward) to identify homogeneous sub-regions from 184 catchments in southeast Australia and forecast low flow characteristics. They found that the best combinations are Ward with squared Euclidean and average with cosine. Later, Cunderlik and Burn^21^ recommended using Mahalanobi's distance since it considers the variance and covariance of variables, which is not obvious with other distances. Shirkhorshidi et al.^22^ compared similarity and dissimilarity measures in clustering various continuous data sets. They employed the Minkowski family, including Euclidean and Manhattan distances and the modified versions of Euclidean distance: average, weighted Euclidean, and chord distance; cosine similarity measure; and Pearson correlation. They concluded that average distance was among the topmost accurate measures for all clustering algorithms.

In south Mediterranean regions, study cases are not so large. Singla et al.^23^ studied the hydrological regimes of 27 river basins in Morocco to assess the impact of climate change on water resources. They applied the regional vector method to outline homogeneous rainfall variability and assess the representativeness and persistence of regional signals. They outline that in the Rif and the Mediterranean Sea, rainfall revealed a trend towards a relative increase since 1980 but a significant decrease in other regions. Monthly and annual discharge analyses showed a decrease since the late 1970s. In 2017, Totz et al.^24^ developed a new cluster-based empirical forecast method (HCA) to predict precipitation anomalies in winter. This method outperformed both statistical and dynamical models over comparable historical periods in the European and Mediterranean regions.

In the south Mediterranean region, Ahattab et al.^25^ utilized morphological parameters and the series of monthly precipitation recorded at 23 rainfall stations (with a common observation period of 15 years) spread across the Tensift watershed (Morocco) to identify four homogeneous clusters that can be considered to exhibit hydrologically similar behaviors and for which the same models for estimating flood peaks can be applied. In Tunisia, few hydrological regionalization studies involving catchment classification were done. Bargaoui et al.^26^ applied the ISODATA method to regionalize 39 Tunisian catchments and assess the centennial flood. The copula model classification based on physiographic and geographic catchment characteristics was later investigated by Gargouri and Bargaoui^27^ to delineate 22 Tunisian catchments in hydro-physiographical regions. They noted that the catchments in the same region are not necessarily geographically contiguous.

Subsequently, At 55 stations of the Tunisian gauging network, Bargaoui and Chebchoub^28^ applied the multifractal analysis of maximum annual flood discharges. They identified a random cascade model after successfully connecting the various statistical moments of the basins' surface discharges through a scale-invariant law.

Next, Cherif and Bargaoui^29^ used HCA to construct a mean regional frequency curve for annual maximum runoffs and applied topographic descriptors for cluster analysis. They utilized Trellis and hierarchical classifications for partitioning with a sample of 40 Tunisian watersheds. Various multidimensional spaces were studied with pairs or triplets of attributes to construct the distance measures. Finally resulting clusters were checked for hydrological homogeneity applying the Hosking and Wallis test. They concluded that global slope index is highlighted as scale factor for flood index.

Then, Cherif and Gargouri^30^ studied the hydrologic behavior of twenty catchments situated on Tunisian ridge. To define hydrologic regions, they used the Hosking and Wallis test and the moving average clustering method related to catchment hydro-geomorphologic attributes^34^. Next, they hold on to regional frequency curves of the maximum specific discharge index.

Later, Kotti et al.^31^ used the regional vector method to divide the study area into six climatically homogeneous subregions after identifying the regional components of the variability of river flows in the Medjerda watershed (the largest river basin in Tunisia). Then they developed regionalized regression models to determine the runoff coefficient and studied the inter-correlations between stations to fill in a series of flow data.

They validated the possibility of estimating runoff at a station based on the maximum rate and the rain from the same station and hydrologic parameters from a neighboring gauging station, with a noticeable improvement in runoff depth values compared to the literature. Recently, Gargouri et al. [32] used Ward's algorithm with Euclidian distance and agglomerative hierarchical clustering to study 22 Tunisian catchments, where the dissimilarity between clusters is calculated in the multidimensional space of geomorphological and physiographical variables. Then, regions homogeneity and consistency are measured by the silhouette index. This study led to three homogeneous regions, performed using a multivariate copula.

Although there has been significant research in PUB applications on Mediterranean cases clustering Tunisian catchments and Sud Mediterranean regions, HCA techniques applications remain limited. More efforts are needed in clustering analysis applications that are still uncommon and underrepresented due to the difficult gauging circumstances and lack of hydrometric data in the region.

Indeed, better understanding of Tunisian catchment’s behavior can be highly valuable for hydrologists in Tunisia, It contributes to the advancement of hydrological modeling, supports decision making processes in water resources management and offers beneficial insights into the hydrological characteristics of other Mediterranean regions, especially the Sud Mediterranean that have climate and agricultural practices similarities.

This study aims to analyze hydrologic similarity between Tunisian ridge catchments based on the HCA algorithm and the homogeneity index of delineated clusters. Several metric distances were applied in the linkage method, and their efficiencies are compared.

(i) To attempt this objective, the following steps will be carried out: applying HCA for Tunisian catchments with similarity distances based on their geo-morphological attributes and hydrometrical signatures.
(ii) Integrating Silhouette index to validate the homogeneity of clusters. Hence, we compare efficiency of all distances to predict the most accurate one.
(iii) Analyzing results to better understand Tunisian catchment’s behaviors.

## Materials and methods

### Clustering approach

HCA is an unsupervised multivariate analysis that classifies the given data into similar, overlapping, or non-overlapping clusters. It has large applications for finding homogeneous clusters of objects based on metric distances between objects. HCA seeks to build a hierarchy of clusters that can be agglomerated or divisive. Agglomerating algorithms merge clusters. On the contrary, divisive algorithms split clusters. Both can be illustrated as a nested sequence or tree diagram, called a dendrogram. It shows the linkage points and clusters that are connected at increasing levels of dissimilarity. The heights of the branch points indicate how similar or different they are from each other; the greater the height, the greater the difference.

In the current study, the HCA algorithm is applied to delineate clusters of similar catchments; we focus on defining the most homogeneous clusters. Homogeneity is defined by the similar hydrodynamic behavior of catchments. Hence, we are seeking the more suitable distance that gives the best similarity in the clusters.

**Figure 1:**
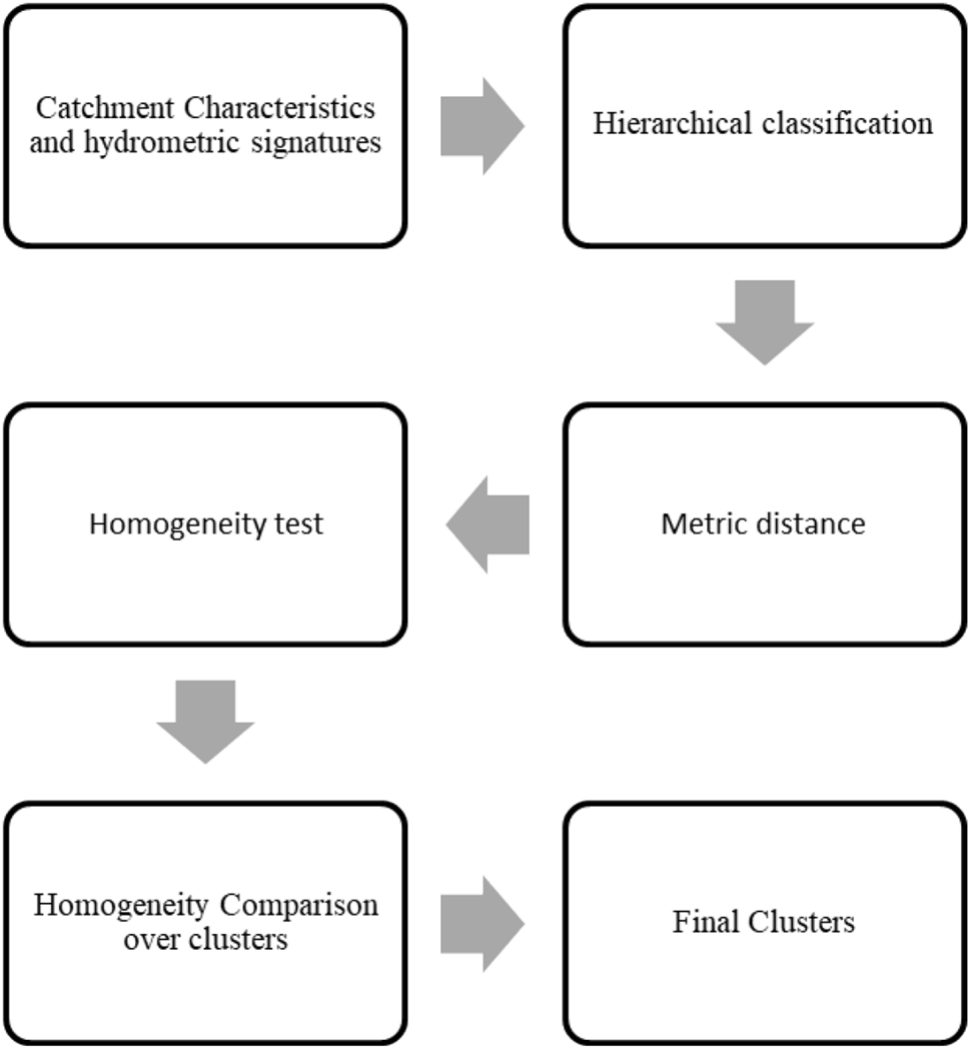
Illustration of the clustering methodology steps.

As a first step, correlations are calculated between all attributes and signatures after their standardization. Then, all specified attributes and signatures are implemented in HCA. Cluster homogeneity is assumed to be ensured; afterward, to validate this hypothesis, the silhouette index is calculated. Each cluster is characterized by its silhouette index, which compares its tightness and separation. It illustrates which feature vectors belong to the cluster and which ones are just in between clusters. Cluster’s silhouette indexes show consistency within clusters and provide a means of assessing cluster quality^32^. They are calculated for each cluster and then compared between all applied similarity distances to outline the best one for the hierarchical clustering approach. The steps of the clustering methodology applied in our current study are summarized in Fig. 1.

### Distance’s equations

Let’s consider a matrix *X* of size $n\times p$: rows are the individuals (*n*), and columns are the variables (*p*) (Eq. 1.)

1 $$X=\left[\begin{array}{ccc}{x}_{1}^{1}& \cdots & {x}_{1}^{p}\\ \vdots & {x}_{i}^{j}& \vdots \\ {x}_{n}^{1}& \dots & {x}_{n}^{p}\end{array}\right]$$

With *x*_*i*_: ith row and *x*_*j*_: jth column.

The distance $d\left({x}_{i},{x}_{j}\right)$ is defined between two vectors *x*_*i*_ and *x*_*j*_* (i, j* = *1…n)* in the *p*-dimensional space ${\mathbb{R}}^{p}$.The distances utilized in this work, for hierarchical analysis, are illustrated in Table 1.

**Table 1:** Distance equations utilized for hierarchical analysis.

Distance	Expression $d\left({x}_{i},{x}_{j}\right)$	Comments
Euclidean	$\sqrt{\left({x}_{i}-{x}_{j}\right){\left({x}_{i}-x\_j\right)}^{t}}$	${x}^{t}$*: transpose vector*
Standardized euclidean	$\sqrt{\left({x}_{i}-{x}_{j}\right){V}^{-1}{\left({x}_{i}-{x}_{j}\right)}^{t}}$	V is the *p-by-p* diagonal matrix whose *j*_*th*_ diagonal element is squared standard deviation;
Chebyshev	${max}_{k}\left\{\left|{x}_{i}^{k}-{x}_{j}^{k}\right|\right\}$	$k=1,\dots \dots \dots \dots ,p$
Cosine	$1-\frac{{x}_{i}{x}_{j}^{t}}{\sqrt{\left({x}_{i}{x}_{i}^{t}\right)\left({x}_{j}{x}_{j}^{t}\right)}}$	${\mathrm{x}}^{\mathrm{t}}$: transpose vector
Correlation	$1-\frac{\left({x}_{i}-\overline{{x }_{i}}\right){\left({x}_{j}-\overline{{x }_{j}}\right)}^{t}}{\sqrt{\left({x}_{i}-\overline{{x }_{i}}\right){\left({x}_{i}-\overline{{x }_{i}}\right)}^{t}\sqrt{\left({x}_{j}-\overline{{x }_{j}}\right){\left({x}_{j}-\overline{{x }_{j}}\right)}^{t}}}}$	_
Hamming	$\left(\#\left({x}_{i}^{k}\ne {x}_{j}^{k}\right)/p\right)$	$k=1,\dots \dots \dots \dots ,p$
Jaccard	$\frac{\left[\left({x}_{i}^{k}\ne {x}_{j}^{k}\right)\cap \left(\left({x}_{i}^{k}\ne 0\right)\cup \left({x}_{j}^{k}\ne 0\right)\right)\right]}{\left[\left({x}_{i}^{k}\ne 0\right)\cup \left({x}_{j}^{k}\ne 0\right)\right]}$	$k=1,\dots \dots \dots \dots ,p$
Spearman	$1-\frac{\left({r}_{i}-\overline{{r }_{i}}\right){\left({r}_{j}-\overline{{r }_{j}}\right)}^{t}}{\sqrt{\left({r}_{i}-\overline{{r }_{i}}\right){\left({r}_{i}-\overline{{r }_{i}}\right)}^{t}\sqrt{\left({r}_{j}-\overline{{r }_{j}}\right){\left({r}_{j}-\overline{{r }_{j}}\right)}^{t}}}}$	*r*_*i*_ and *r*_*j*_ are the coordinate-wise rank vectors of *x*_*i*_ and *x*_*j*_

### Performance criteria (Silhouette index) and cluster’s homogeneity validation

One of current study objectives is to assess and compare the efficiency of metric distances in the HCA approach. It is crucial to confirm that delineated clusters, really reflect hydrological homogeneity.

The Silhouette index describes each cluster by contrasting its tightness and separation to assess homogeneity. It illustrates which feature vectors belong to their cluster, and which ones are just in between clusters. Cluster’s Silhouettes are plotted in a chart showing consistency within clusters and providing assessing cluster quality^32^.

For each feature vector *x*_*i*_, the corresponding Silhouette index *s(i)* is defined as:

2 $$s\left(i\right)=\frac{b\left(i\right)-a\left(i\right)}{max\left[a\left(i\right),b\left(i\right)\right]}$$

where, for a given *x*_*i*_ belonging to cluster A (with ) and a distance *d* (.,.),

3 $$a\left(i\right)=\frac{1}{Card\left(A\right)-1}\sum_{\begin{array}{c}{x}_{j}\in A\\ i\ne j\end{array}}d\left({x}_{i},{x}_{j}\right)$$

4 $$b\left(i\right)={min}_{A\ne C}\frac{1}{Card\left(C\right)}\sum_{\begin{array}{c}{x}_{k}\in C\\ i\ne k\end{array}}d\left({x}_{i},{x}_{k}\right)$$

where is the cardinal number. It is thought as an equivalence class of sets. *a* (*i*) is the average distance from the *i*th feature vector to all other feature vectors in the cluster *A*; *b*(*i*) is the minimum average distance from the *i*th feature vector to all the feature vectors in another cluster *C*. From this equation it follows that *-1* ≤ *s*(*i*) ≤ *1*. If *s*(*i*) is large thus the *i*th feature vector is well assigned to the cluster. On the other hand, when *s*(*i*) is close to -1 the *i*th feature vector is not well classified.

The closer Silhouette index approaches to 1, the better cohesion and separation are^33^.

Therefore, it is applied in this study to evaluate and compare clustering approaches, Silhouette indexes are calculated for each catchment in the cluster, and then their averages are deduced. A positive value reveals that the catchment is well matched to its cluster. A negative one means that the catchment is not in the right cluster, so it could be moved to the more closely related one^34^.

Hence a high Silhouette index demonstrates that the classified feature vector (catchment) is well pooled and poorly matched to neighboring clusters. If the Silhouette index value is close to (-1), it means that the individual is not in the right cluster^39^.

## Study case

In current research we compared the use of hierarchical classification with several distances specified in Table 1 (see § 2.2) to delineate catchments into hydrological regions. Silhouette indexes are then calculated for each catchment in each delineated cluster to define the best distance giving the higher homogeneity for clusters (regions). Hence, MATLAB software package is utilized.

**Figure 2:**
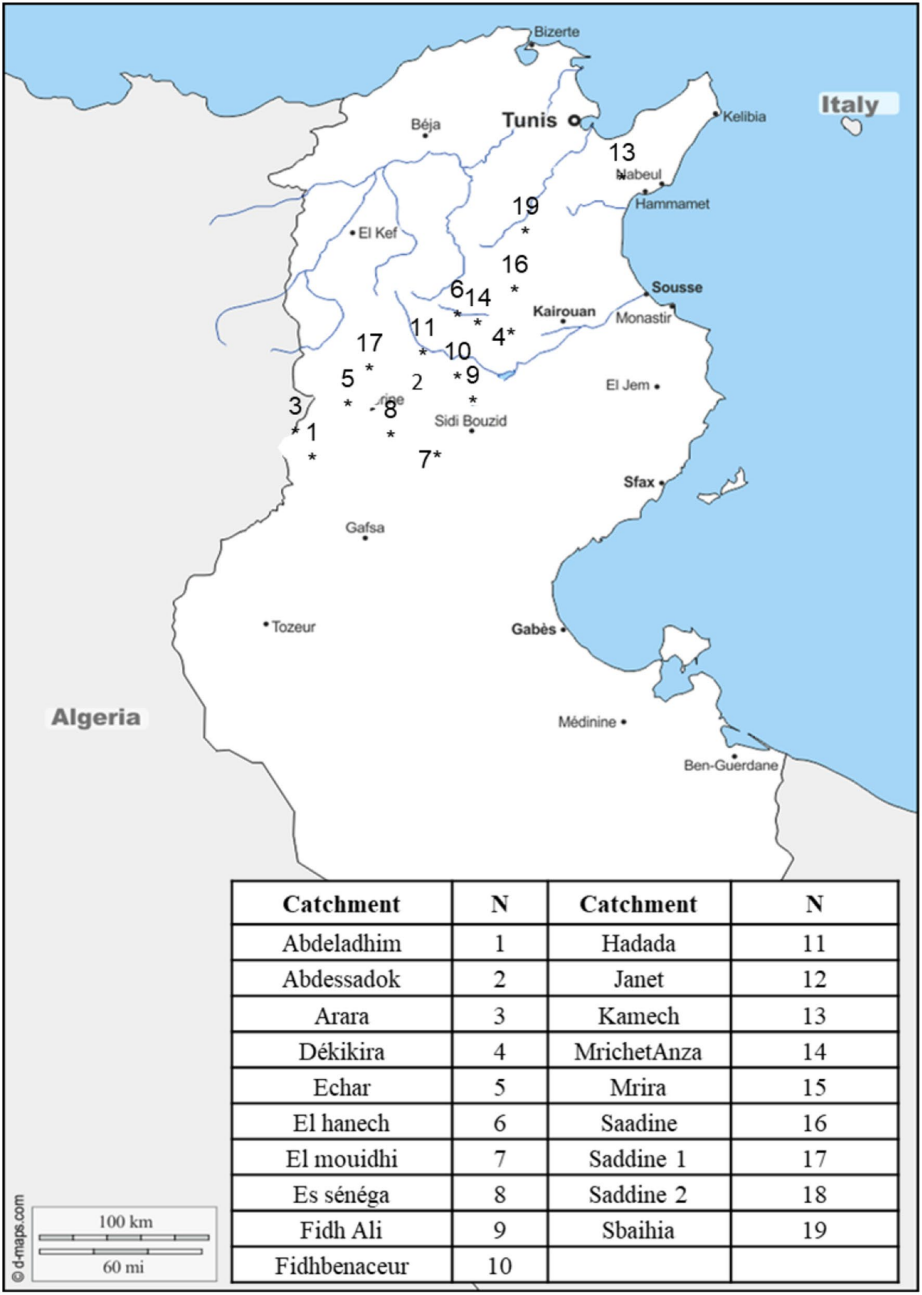
Tunisian Hydrometrical network considered in this study (map from https://d-maps.com, opensource, and modified by QGIS: GNU General Public License, version 2.0).

We considered nineteen 19 catchments situated in the Tunisian ridge and monitored since 1992, controlled by headwater dams. Latitudes vary between 35°N and 37°N; longitudes from 8°E and 11°E, areas range from 1 km^2^ to 10 km^2^ and annual average rainfall vary between 280 and 500 mm, these catchments are in a semi-arid zone. These catchments are little permeable to impermeable and have fairly high too high reliefs that promote rapid runoff. The rain gauge network is composed of 19 gauges Fig. 2, located at each headwater dam^35^.

Two data sets (Catchment’s attributes and streamflow signatures) are utilized, with HCA algorithm, to delineate homogeneous regions. The first set illustrates physiographical catchment’s attributes and are selected because they can predetermine hydrological behavior [37; 5; 38], it is hold in Table A.1 of the appendices and is composed of: Latitude (LatN); longitude (LongE); area (A); Perimeter (P); specific denivelation (D_S_); global slope index (I_s_); Gravellus Index (I_G_); the percentage of path (Pp); the percentage of forest cover (Pf); the percentage of cereal culture area (Pc); the percentage of arboriculture area (Pa); the percentage of area affected by anti-erosive practices (Aae)).

The second set is hold in the Table A.2 of the appendices and summarizes the hydrometrical signatures defined as:

- maximum rainfall intensity (I_max_).
- rainfall duration (D), runoff depth.
- Runoff depth (R),: runoff volume from a drainage basin, divided by its area, in a specified time expressed in mm.
- Hydrograph time to peak (tp): the increase time of hydrograph.
- Hydrograph base time (tb): time between the begin and the end of the hydrograph.
- Infiltration index (ϕ): average rate of infiltration derived from a time intensity graph of rainfall in such a manner that the volume of rainfall in exceedance of this rate will equal the volume of storm runoff^38^.
- Runoff coefficient (Cr): ratio of runoff depth to precipitation depth.
- Average discharge (Q_mean_): average daily runoff.
- Specific Maximum discharge (QS_max_): maximum discharge divided by the catchment area.

These signatures quantify the hydrologic response and provide insight into the functional behavior of the catchment^37^. They are included to support the hypothesis of hydrological homogeneity. Tables A.1 and A.2 holds also, specific statistics such as mean values, standard deviation, minimum values, and maximum values, which are denoted as Min, Max, μ and σ respectively. All data come from hydrological reports of the Tunisian Water Resources Division (DGRE).

## Results and discussion

As a first step, correlations are calculated between all attributes and hydrometrical signatures after their standardization (Table 2a, b). Ranging between − 0.7 and 1, they reveal that geo-morphological attributes are closely to slightly connected with flow signatures and rainfall descriptors, with inter-correlations varying between − 0.5 and 0.8. Hence, watersheds have hydrological behaviors influenced by their geomorphology. This result is in accordance with the work of Kotti et al.^35^ in their study of the Medjerdah watershed, where they deduce that the flows are a relative response to different factors (watershed size, relief, geology, soils, and vegetation cover).

**Table 2:**
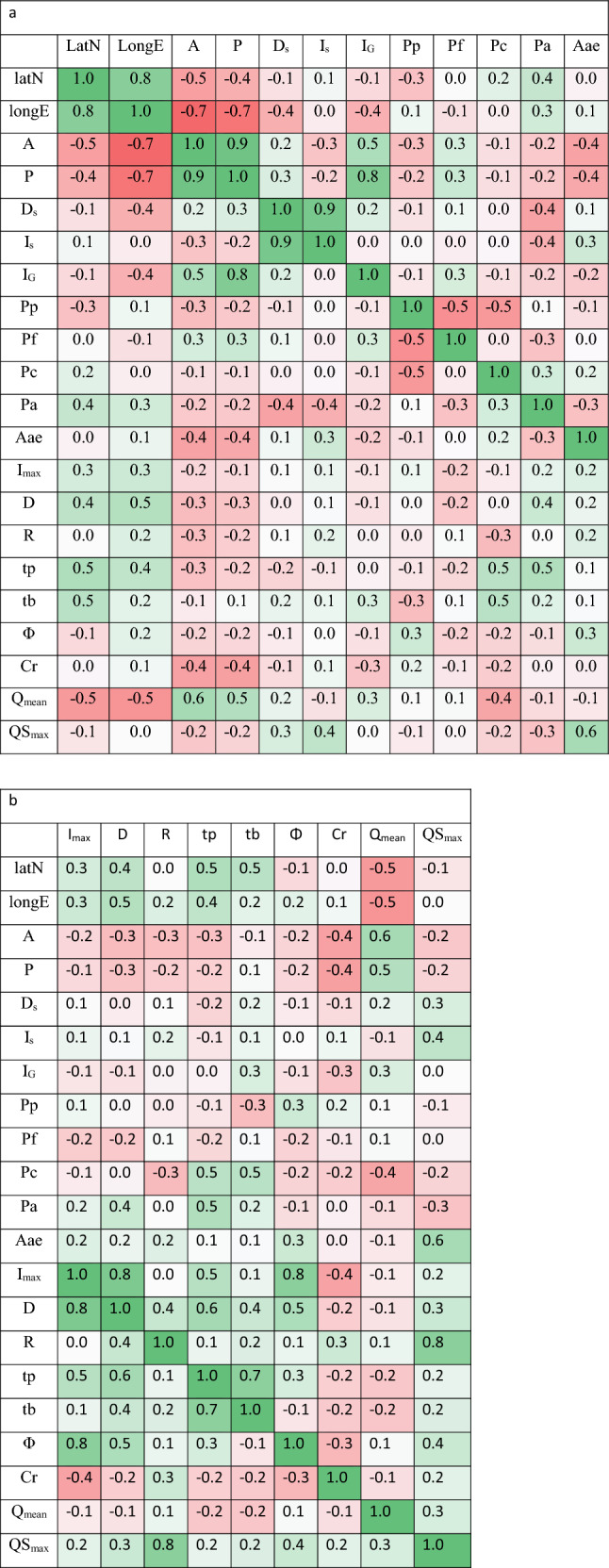
Correlations between attributes and hydrometrical signatures.

Next, HCA is applied with distances calculated from geomorphologic attributes and hydrometrical signatures (previously defined) to delineate clusters of catchments with similar behavior (homogeneous clusters). Hence, we search to outline the best distance involving the most homogeneous clusters. To attempt this objective, all distance similarities previously cited in Table 1 are applied to catchment attributes and signatures.

Due to the limited total number of catchments. They are divided into two clusters with dendrogram agglomeration, and clusters are concatenated graphically. The level of cluster homogeneity is then determined by computing silhouette indices.

Clustering results are summarized in Table 3. This table reveals that catchments 7, 8, and 9 are consistently in the same pool, highlighting a persistent similarity regardless of the distance. Average silhouette values and catchment partitioning for each distance are displayed in Table 4, which indicates that city-block, Hamming, Spearman, and Jaccard distances provide an equal distribution of catchments.

**Table 3:** Clustering results of all metric distances (watershed membership to each cluster).

		Distances								
		Euclidean	Seuclidean	Cityblock	Cheybychev	Cosine	Correlation	Hamming	Spearman	Jaccard
Catchment N	1	1	1	2	2	1	1	2	2	2
	2	2	2	1	2	2	2	2	1	2
	3	1	1	2	2	1	1	1	2	1
	4	2	2	1	2	2	2	2	1	2
	5	1	1	2	2	1	1	1	2	1
	6	2	2	2	2	2	2	1	1	1
	7	2	2	1	2	2	2	1	1	1
	8	2	2	1	2	2	2	1	1	1
	9	2	2	1	2	2	2	1	1	1
	10	2	2	1	2	2	2	2	1	2
	11	1	1	2	2	1	2	1	2	1
	12	1	1	2	2	1	1	1	2	1
	13	2	2	1	2	2	2	2	1	2
	14	2	2	2	2	1	2	2	2	2
	15	1	1	2	2	1	1	2	2	2
	16	2	2	1	1	2	2	2	2	2
	17	2	2	1	1	2	2	2	1	2
	18	1	1	2	2	1	1	1	2	1
	19	2	2	2	2	2	2	2	2	2

**Table 4:** Total number of catchments in each cluster and average silhouette index.

	Cluster 1		Cluster 2	
	Catchment number	Average silhouette index	Catchment number	Average silhouette index
Euclidean	7	0.2309	12	0.0738
Seuclidean	7	0.2309	12	0.0738
Cityblock	9	0.1226	10	0.1465
Cheybychev	2	− 0.0229	17	0.1122
Cosine	8	0.2838	11	0.2145*
Correlation	6	0.4186*	13	0.1778
Hamming	9	0.0475	10	0.0005
Spearman	9	0.2685	10	0.1661
Jaccard	9	0.0475	10	0.0005

*maximum value.

All distances indicate positive average Silhouette indexes (ASI) values for both clusters, ranging from 0.04 to 0.418 for the first cluster and from 0.001 to 0.188 for the second one. So, catchments in first cluster indicate a greatest consistency (homogeneity).

Hence, we deduce that Correlation distance provides the best consistent groups with ASI values of 0.42 and 0.18. The first cluster is composed of 32% of total catchments when the second one implies 68% of them. It is followed by Cosine and Spearman distances with Silhouette indexes respectively of [0.28; 0.21] and [0.27; 0.17]. Euclidean and Seuclidean reveals similar results. Cityblock, Hamming and Jaccard distances produce a nearly equal distribution (with 9 and 10 catchments in each cluster). However, Hamming and Jaccard distances display clusters with the lowest similarities for which Silhouette indexes are equal respectively to [0.048 and 0.0005]. We conclude that with these distances, catchments of the first cluster are more hydrometrically similar.

**Figure 3:**
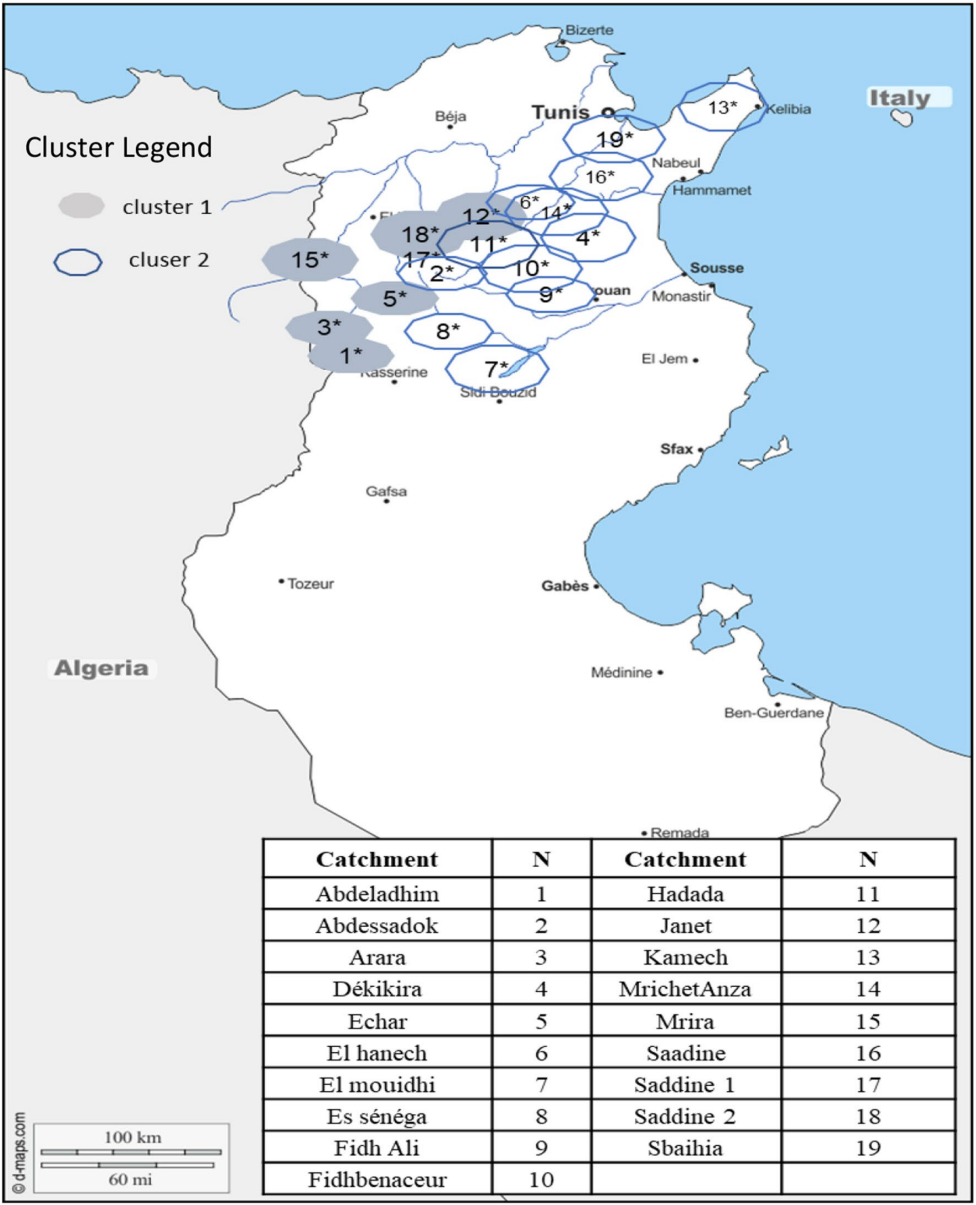
The Clusters achieved with the correlation distance (map from https://d-maps.com, opensource, and modified by QGIS: GNU General Public License, version 2.0).

Correlation distance reveals the most homogeneous clusters. Figure 3 holds on catchments belonging to each cluster. It is worth noting that the catchments in a same cluster are not necessarily geographically contiguous; in effect, the geographical proximity of the catchments is not a guarantee of their hydrological similarity^39^. This result is in accordance with the one described by Gargouri-Ellouze and Bargaoui^35^.

Therefore, we sign that the distance selection could improve accuracy of the clustering method and the hydrological homogeneity in the clusters: This outcome must be considered when dealing with regionalization studies in south Mediterranean regions.

This effect is in accordance with Totz et al.^24^ study, in which they developed a new cluster-based empirical forecast method (HCA), to predict winter precipitation’s anomalies in European and Mediterranean regions.

Their method achieves a higher skill than other empirical methods used in the past such as the multi-regression model developed by Eden et al.^40^ or the CCA-based algorithm applied by Barnston et al.^41^.

Catchments within each cluster, statistics of physiographical attributes and hydrometrical signatures are summarized below (Table 5), as well as the ratios of means (ρ) in each cluster for all attribute and signature. ρ = µ_1_/µ_2_ where µ_1_ and µ_2_ are means of (attribute or signature) in respectively the first and the second cluster.

**Table 5:** Statistics of cluster’s attributes and signatures derived from Correlation distance.

		Cluster 1				Cluster 2				ρ
		Min	Max	µ	σ	Min	Max	µ	Σ	
Physiographical attributes	A (km^2^)	5.2	9.2	6.8	1.3	1.6	4.7	3.1	0.9	2.2
	P (m)	11.6	16.8	13.8	2.0	5.5	9.9	8.0	1.4	1.7
	D_S_ (m)	86.7	207.5	135.9	43.3	62.6	224.3	133.6	63.3	1.0
	I_s_ (m/km)	35	78	53.0	17.7	37	128	74.9	30.2	0.7
	I_G_	1.3	1.8	1.5	0.2	1.2	1.4	1.3	0.1	1.2
	Pp (%)	0	39	19.7	13.4	0	84	34.5	25.7	0.6
	Pf (%)	0	50	24	21.9	0	57	7.5	17.9	3.2
	Pc(%)	0	87	41.5	31.7	0	76	36.8	28.9	1.1
	Pa (%)	0	4	1.2	1.6	0	8	1.7	2.7	0.7
	Aae (%)	0	5	3.3	2.6	0	50	12.7	14.4	0.3
Hydrometric signatures	I_max_ (mm/h)	15	27	21.5	7.9	16	37.4	26.3	6.4	0.8
	D (min)	24	37	30.7	10.5	20	80.4	42	16	0.7
	R (mm)	808	2454	1508	726	411	6968	2237	1584	0.7
	tp (min)	38	127	84.0	37.8	54.0	217.5	102.9	54.0	0.8
	tb (min)	143	558	307.8	161.4	122.0	523.7	271.5	135.1	1.1
	Φ(mm/h)	15	27	21.3	7.7	16.1	35.4	25.4	5.6	0.8
	Cr	0.0	0.2	0.1	0.1	0.1	0.5	0.2	0.1	0.7
	Q_mean_ (m^3^/s)	0.5	2.7	1.3	0.9	0.1	1.6	0.7	0.5	1.8
	QS_max_ (m^3^/s:km^2^)	0.3	0.9	0.5	0.3	0.1	2.7	0.9	0.8	0.6

µ: mean; σ: standard deviation.

We notice that the first cluster contains larger averages runoffs and areas., indicating wetter and larger catchments. With mean runoff rates twice as high as the second cluster (ρ = 1.8). The Percentage of forest data (Pf) are three times higher as those recorded in the second pool revealing that it greatly controls clustering results. Hence large catchments with important forest covers have similar runoffs indicating similar hydrological behaviors. This outcome can be valuable to neighboring countries within the same climate (Mediterranean regions) and then extended to other anthropogenic indexes.

**Figure 4:**
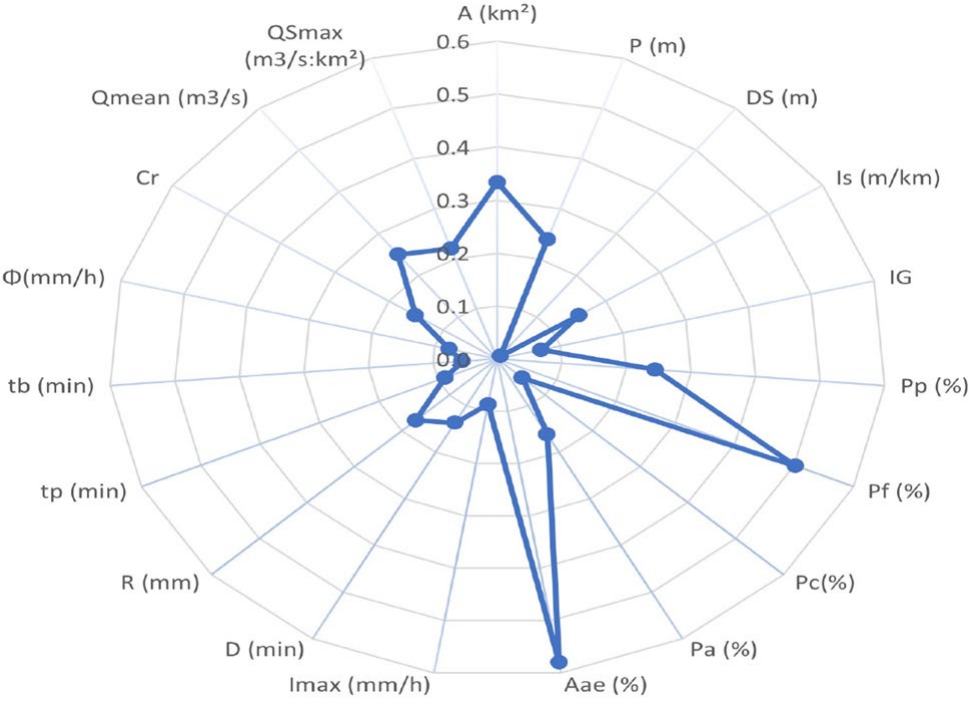
Variation of log absolute ratios of means attributes (ρ = µ_1_/µ_2_).

Figure 4 holds on log absolute values of these ratios which can enlighten us about the most discriminant attributes and signatures. Zero indicates similar averages between the two clusters and the further from zero, the more discriminating the attribute is (resp. signature). It demonstrates three discriminant attributes: the percentage of area affected by anti-erosive practices (Aae) which is the most significant, followed by the percentage of forest cover (Pf) and catchment’s areas (A). Cluster1 is made of catchments with important forest cover, weak anti-erosive practices, and greater than 5 km^2^. Cluster 2 is constituted by catchments with weak forest cover, important anti-erosive practices and smaller than 5km^2^. Each cluster has its own specific hydrological behavior. This point will have to be respected in catchment modeling and runoff forecasting.

These results are in harmony with reviews detailing specific aspects of the hydrology of Mediterranean catchments such Mediterranean forest impact on catchment responses^42^, the dryland hydrology^43^ and erosion processes^44,45^.

Finally, the delineation approach applied in current work reveals that distance between geomorphologic attributes and hydrometric signatures impacts the HCA delineation results so the hydrological pooled regions. This study can be considered an example case for Sud Mediterranean basins that can be extrapolated with other neighboring data as Algerian catchments.

## Conclusion

The current research described in this paper explores the use of unsupervised HCA in clustering Tunisian catchments which is applied with various distances calculated from associated attributes and signatures. Nineteen catchments are involved, and nine metric distances are explored to identify the most hydrologically similar clusters. Nineteen geomorphologic attributes and hydrometrical signatures (rainfall and flow signatures) are applied in this work to calculate diverse metric distances in HCA considered for delineating homogeneous clusters.

After performing the clustering step, Silhouette indexes are calculated for each cluster. They reveal that Correlation distance gives widely the most homogeneous clusters, compared with the other distances. It gives two clusters, not equally scattered (32% and 68% of total catchments) with average Silhouette indexes equal to 0.42 and 0.18.

Statistics show that the percentage of area affected by anti-erosive practices, the percentage of forest cover and catchment’s area are the most discriminant attributes. However, hydrometrical signatures appear to be not relevant. This partitioning allowed to highlight two different hydrological behaviors which must be considered in modeling and/or forecasting.

Finally, these results can be helpful in regionalization strategy to calibrate hydrological models in south Mediterranean regions when the shortage of hydrometrical data is an occurring problem. They can be considered promising by the way that they can be advantageous in some cases of hydrologic predictions without need of heavy hydrologic information in ungauged catchments. Our study can be considered as a sample of Sud Mediterranean basins that can be extrapolated with data of other neighboring regions such as Algerian catchments.

## Supplementary Information

Supplementary Information.

## Data Availability

All data generated or analyzed during this study are included in the published article “ Gargouri-Ellouze E. & Bargaoui Z. Investigation with Kendall plots of infiltration index–maximum rainfall intensity relationship for regionalization. *Physics and Chemistry of the Earth,* Parts A/B/C, **34**(10–12), 642–653. (2009) ” and its supplementary information files.
