# Supplementary material for: Flow signatures and catchment’s attributes for HCA clustering in a hydrologic similarity assessment (Tunisian case)
Source: Sci Rep. 2023 Jul 26;13:12144. doi: 10.1038/s41598-023-38608-6 (PMC10371992; doi:10.1038/s41598-023-38608-6)
Supplement: Supplementary file 1 — Supplementary Information. [file 41598_2023_38608_MOESM1_ESM.docx]

**Appendices**

Table A.1.1 : Geomorphological attributes

| **Catchment** | **N** | **LatN** | **LongE** | **A**  **(km²)** | **P**  **(km)** | **Ds**  **(m)** | **I_s_**  **(m/km)** | **I_G_** | **Pp**  **(%)** | **Pf (%)** | **Pc**  **(%)** | **Pa**  **(%)** | **Aae (%)** |
| --- | --- | --- | --- | --- | --- | --- | --- | --- | --- | --- | --- | --- | --- |
| Abdeladhim | 1 | 35°13'01" | 8°33'02" | 6,42 | 11,58 | 114,0 | 45 | 1,28 | 10 | 42 | 54 | 1 | 5 |
| Abdessadok | 2 | 35°40'52" | 9°14'49" | 3,07 | 7,95 | 224,3 | 128 | 1,27 | 37 | 0 | 51 | 1 | 20 |
| Arara | 3 | 35°22'09" | 8°24'25" | 7,08 | 13,85 | 207,5 | 78 | 1,46 | 25 | 37 | 19 | 0 | 5 |
| Dékikira | 4 | 35°53'04" | 9°40'53" | 3,07 | 7,65 | 64,8 | 37 | 1,22 | 54 | 5 | 25 | 8 | 0 |
| Echar | 5 | 35°33'11" | 8°40'45" | 9,17 | 15,5 | 106,0 | 35 | 1,43 | 21 | 0 | 0 | 0 | 0 |
| El hanech | 6 | 36°04'01" | 9°26'55" | 3,95 | 9,55 | 206,7 | 104 | 1,35 | 54 | 0 | 0 | 2 | 5 |
| El mouidhi | 7 | 35°14'24" | 9°50'42" | 2,66 | 7,7 | 70,1 | 43 | 1,32 | 84 | 0 | 0 | 0 | 5 |
| Es sénéga | 8 | 35°29'21" | 9°06'18" | 3,63 | 8,48 | 165,8 | 87 | 1,25 | 39 | 0 | 40 | 2 | 20 |
| Fidh Ali | 9 | 35°42'04" | 9°36'13" | 4,12 | 8,6 | 77,1 | 38 | 1,19 | 47 | 0 | 5 | 0 | 5 |
| Fidhbenaceur | 10 | 35°43'26" | 9°35'20" | 1,69 | 5,75 | 71,5 | 55 | 1,24 | 20 | 0 | 72 | 1 | 50 |
| Hadada | 11 | 35°50'25" | 9°07'42" | 4,69 | 9,9 | 203,6 | 94 | 1,28 | 0 | 0 | 76 | 0 | 0 |
| Janet | 12 | 35°52'16" | 9°11'35" | 5,21 | 12,95 | 152,9 | 67 | 1,59 | 39 | 0 | 61 | 2 | 5 |
| Kamech | 13 | 36°52'18" | 10°52'08" | 2,45 | 7,25 | 62,6 | 40 | 1,3 | 16 | 0 | 74 | 7 | 0 |
| MrichetAnza | 14 | 36°05'37" | 9°35'41" | 1,58 | 5,5 | 90,5 | 72 | 1,23 | 48 | 0 | 32 | 1 | 0 |
| Mrira | 15 | 35°36'34" | 8°28'37" | 6,13 | 12,3 | 86,7 | 35 | 1,39 | 0 | 15 | 87 | 4 | 0 |
| Saadine | 16 | 36°06'55" | 9°56'36" | 2,72 | 8,28 | 153,4 | 93 | 1,4 | 0 | 36 | 0 | 0 | 20 |
| Saddine 1 | 17 | 35°47'49" | 9°03'58 " | 3,84 | 9,7 | 207,7 | 106 | 1,39 | 50 | 0 | 51 | 0 | 20 |
| Saddine 2 | 18 | 35°47'53" | 9°04'42 " | 6,53 | 16,8 | 148,2 | 58 | 1,84 | 23 | 50 | 28 | 0 | 5 |
| Sbaihia | 19 | 36°29'43" | 10°12'31" | 3,24 | 7,38 | 138,6 | 77 | 1,15 | 0 | 57 | 52 | 0 | 20 |
| Min |  |  |  | 1,58 | 5,5 | 62,6 | 35 | 1,15 |  |  |  |  |  |
| Max |  |  |  | 9,17 | 16,8 | 224,3 | 128 | 1,84 |  |  |  |  |  |
| µ |  |  |  | 4,3 | 9,8 | 134,3 | 68,0 | 1,3 |  |  |  |  |  |
| σ |  |  |  | 2,0 | 3,2 | 56,5 | 28,3 | 0,2 |  |  |  |  |  |

A: area; P: Perimeter, I_s_: global slope index; D_S_: specific denivelation; Pp: percentage of path; Pf: percentage of forest cover; Pc: percentage of cereal culture area ; Pa : percentage of arboriculture area; Aae: percentage of area affected by anti-erosive practices; µ average ; σ : standard deviation .

Table A1.2: Rainfall descriptors and stream flow signatures

| Catchment | N | I_max_ (mm/h) | D (min) | R  (mm) | t_p_ (min) | t_b_ (min) | Φ  (mm/h) | Cr | Q_mean_ (m^3^/s) | QS_max_ (m^3^/s/km^2^) |
| --- | --- | --- | --- | --- | --- | --- | --- | --- | --- | --- |
| Abdeladhim | 1 | 26 | 37 | 1394 | 90.9 | 205.7 | 26.354 | 0.179 | 0.619 | 0.3 |
| Abdessadok | 2 | 29 | 42 | 2118 | 67.7 | 250.1 | 29.035 | 0.112 | 0.869 | 1.6 |
| Arara | 3 | 16 | 29 | 2104 | 37.6 | 258.1 | 15.532 | 0.19 | 2.733 | 0.9 |
| Dékikira | 4 | 24 | 37 | 3110 | 132.3 | 156.9 | 23.506 | 0.23 | 1.634 | 0.5 |
| Echar | 5 | 27 | 32 | 808 | 60.6 | 143.4 | 26.638 | 0.071 | 2.363 | 0.6 |
| El hanech | 6 | 30 | 45 | 1329 | 74.5 | 266.7 | 19.27 | 0.11 | 0.354 | 0.2 |
| El mouidhi | 7 | 19 | 29 | 2458 | 69.8 | 169 | 30.355 | 0.183 | 0.759 | 0.8 |
| Es sénéga | 8 | 30 | 47 | 2362 | 58.4 | 180 | 30.455 | 0.169 | 0.881 | 0.6 |
| Fidh Ali | 9 | 30 | 49 | 2287 | 66.9 | 155.8 | 29.542 | 0.112 | 0.905 | 0.5 |
| Fidhbenaceur | 10 | 26 | 39 | 1773 | 103.5 | 228.1 | 25.667 | 0.151 | 0.778 | 1.5 |
| Hadada | 11 | 19 | 27 | 1370 | 78.7 | 205.3 | 18.511 | 0.153 | 0.581 | 0.3 |
| Janet | 12 | 18 | 29 | 2454 | 127.3 | 557.5 | 17.763 | 0.165 | 0.542 | 0.5 |
| Kamech | 13 | 37 | 80 | 1773 | 217.5 | 523.7 | 27.163 | 0.082 | 0.178 | 0.3 |
| MrichetAnza | 14 | 16 | 20 | 411 | 54 | 122 | 16.061 | 0.463 | 0.072 | 0.1 |
| Mrira | 15 | 15 | 24 | 1416 | 112.6 | 454 | 15.127 | 0.103 | 0.568 | 0.3 |
| Saadine | 16 | 24 | 54 | 6968 | 101.5 | 403.1 | 23.776 | 0.332 | 0.986 | 2.7 |
| Saddine 1 | 17 | 35 | 53 | 2128 | 210.4 | 506.8 | 35.421 | 0.129 | 1.289 | 1.8 |
| Saddine 2 | 18 | 27 | 33 | 872 | 75 | 227.8 | 26.578 | 0.046 | 0.97 | 0.3 |
| Sbaihia | 19 | 22 | 24 | 997 | 102.1 | 361.8 | 21.972 | 0.089 | 0.214 | 0.1 |
| Min |  | 15 | 20 | 411 | 37.6 | 122 | 15.127 | 0.046 | 0.072 | 0.1 |
| Max |  | 37 | 80 | 6968 | 217.5 | 557.5 | 35.421 | 0.463 | 2.733 | 2.7 |
| Mean (µ) |  | 25 | 38 | 2007 | 96.9 | 282.9 | 24.143 | 0.162 | 0.910 | 0.7 |
| STD (σ) |  | 6 | 14 | 1384 | 48.3 | 140.3 | 5.818 | 0.098 | 0.692 | 0.7 |

I_max_: maximum rainfall intensity; D: rainfall duration; R: runoff depth; t_p_: hydrograph time to peak; t_b_: hydrograph base time; phi φ: infiltration index; Cr: runoff coefficient; Q_mean_: mean discharge; QS_max_ specific: maximum specific discharge.
